# CsWRKY25 Improves Resistance of Citrus Fruit to *Penicillium digitatum via* Modulating Reactive Oxygen Species Production

**DOI:** 10.3389/fpls.2021.818198

**Published:** 2022-01-10

**Authors:** Wenjun Wang, Ting Li, Qi Chen, Shixiang Yao, Lili Deng, Kaifang Zeng

**Affiliations:** ^1^College of Food Science, Southwest University, Chongqing, China; ^2^Research Center of Food Storage & Logistics, Southwest University, Chongqing, China

**Keywords:** citrus, CsWRKY25, *Penicillium digitatum*, ROS, disease resistance

## Abstract

WRKY transcription factors (TFs) play crucial roles in the regulation of biotic stress. Citrus is the most productive fruit in the world. It is of great value to investigate the regulatory molecular mechanism of WRKYs in improving disease resistance. In this research, the transcription level of *CsWRKY25* was upregulated in *P. digitatum* infected citrus peel, and CsWRKY25 activated the expression of three target genes (*RbohB, RbohD*, and *PR10*). Besides, the *Agrobacterium*-mediated transient overexpression of *CsWRKY25* has also been shown to enhance resistance to *P. digitatum* in citrus, and caused the accumulation of hydrogen peroxide and lignin. The accumulation of ROS also activated the antioxidant system, the catalase (*CAT*), peroxidase (*POD*), and cinnamyl alcohol dehydrogenase (*CAD*) genes were significant upregulated, leading to activation of antioxidant enzymes. In addition, the up-regulated expression of *MPK5* and *MPK6* genes suggested that the regulatory role of CsWRKY25 might be related to the phosphorylation process. In conclusion, CsWRKY25 could enhance the resistance to *P. digitatum via* modulating ROS production and PR genes in citrus peel.

## Introduction

Plants have developed an effective array of physical and chemical based defenses designed to stop the invasion of harmful microorganisms during evolution (Zhou and Chai, 2008), among them, reactive oxygen species (ROS) is qualified as the first line of defense against pathogen infection (Pitino et al., 2015). ROS is an important signaling molecule in plant inherent immune system, that can strengthen the cell wall structure by promoting the synthesis and accumulation of lignin, and then inhibit the pathogen infection. But the process must be tightly regulated to avoid the oxidative stress caused by excessive accumulation of ROS, which could also be toxic to plants. And the antioxidant compounds and enzyme systems in plants are therefore developed to maintain the homeostasis of reactive oxygen species. The major antioxidant enzymes are catalase (CAT), peroxidase dismutase (SOD) glutathione peroxidase (GPX), peroxidase (POD), and glutathione-S-transferase in plants (Chae et al., 1999; Matés et al., 1999). H_2_O_2_ can activate the mitogen-activated protein kinase (MAP kinase, MAPK) to amplify the signal transduction pathway and activate the transcription factors (TFs) which promote the expression of downstream target genes, and then increase the disease resistance of plants (Danquah et al., 2014; Gao et al., 2018).

TFs are crucial in regulating plant biotic stress, WRKYs are one of the most important and largest TFs in disease resistance response of plants (Lei et al., 2020; Rushton et al., 2010). WRKYs are involved in two layers of immunity, PAMP-triggered immunity (PTI) and effector-triggered immunity (ETI) (Eulgem and Somssich, 2007). WRKYs contain at least one WRKY domain (WRKYGQK motif), and could bind to the W-box [(T)(T)TGAC(C/T)] (Liu et al., 2016; Llorca et al., 2014; Rushton et al., 2010). As demonstrated by previous reports, WRKY70 is involved in SA-mediated disease resistance (Deng et al., 2020; Li et al., 2004). *Arabidopsis thaliana* AtWRKY7, AtWRK22, AtWRKY33, and AtWRKY54 have also been verified to be directly involved in the resistance against fungi (Birkenbihl et al., 2012; Hsu et al., 2013; Kim et al., 2006; Li et al., 2017). Likewise, overexpression of the *Populus trichocarpa PtrWRKY73* or strawberry *FaWRKY1* in *Arabidopsis* can enhance the resistance against *Pseudomonas syringae* (Duan et al., 2015; Encinas-Villarejo et al., 2009); overexpression of *OsWRKY71* can improve the resistance to *Xanthomonas oryzae pv. Oryzae* in rice (Liu et al., 2007); and overexpression of *GhWRKY44* upregulates the expression of *PRs* in cotton (Li et al., 2015).

Citrus is the most productive fruit in the world, but *Penicillium digitatum* (*P. digitatum*) causes severe losses of citrus fruit, accounting for about 90% of total losses (Droby et al., 2002). But there are still few reports on WRKYs in regulating biotic stress of citrus fruit. In our previous study on citrus fruit, CsWRKY65 and CsWRKY70 were found to be important regulators of plant resistance against *P. digitatum* (Deng et al., 2020; Wang et al., 2021). And CsWRKY22 in sweet orange could promote the expression of *CsLOB1* and enhance the susceptibility to canker (Long et al., 2021). Thus, the isolation and cloning of citrus disease resistance WRKY TFs genes and the exploration of molecular mechanism therein are of great significance. *CsWRKY25* was identified in response to *P. digitatum* (Deng et al., 2018). The present study was undertaken to investigate the molecular characterization of CsWRKY25, the effect of CsWRKY25 on citrus resistance and the possible regulatory mechanisms on reactive oxygen species, and the related enzymatic antioxidant system.

## Materials and Methods

### Pathogen, Fruit and Treatments

*Penicillium digitatum* spores were obtained by cultivating on potato dextrose agar (PDA) plates (Jeong et al., 2016). Spores were titrated with a hemacytometer. The intact citrus fruit [*Citrus sinensis (L.)* Osbeck cv. Jincheng 447#] were harvested from an organic orchard in Chongqing, China, selected and surface-disinfected with 2% (v/v) sodium hypochlorite as described previously (Wang et al., 2021).

### RNA Extraction, Gene Isolation, and Sequence Analysis

Total RNA was extracted from citrus fruit materials using a cetyltrimethylammonium bromide (CTAB) method with a few modifications (Moore et al., 2005). RNA concentration and quality was monitored with a Nanodrop 1000 spectrophotometer (Thermo scientific, United States) and assessed by 1% agarose electrophoresis. The PrimeScript™ RT reagent Kit with gDNA Eraser (RR047Q, Takara, Japan) was employed to synthesize the cDNA. The coding DNA region of *CsWRKY25* was cloned. Multiple alignment was performed by Tbtools (Chen et al., 2018), and phylogenetic tree of WRKY25 protein was generated by MEGA7.

### qRT-PCR

The gene expression was detected by qRT-PCR. The iTaq™ Universal SYBR Green Supermix and a CFX384 Touch Real-Time PCR Detection System (BIO-RAD, United States) were used. *Citrus sinensis* actin gene (LOC102577980) was employed as endogenous a reference gene. The 2^–ΔΔ*CT*^ formula was calculated as the relative expression for data analyses (Livak and Schmittgen, 2001).

### Genomic DNA Extraction, Promoter Isolation and Analysis

Genomic DNA was extracted from tender leaves of citrus tree using a DNAsecure Plant Kit (Tiangenbiotech, Beijing, China). Promoter fragments of the *CsRbohB*, *CsRbohD* and *CsPR10* genes were amplified by PCR. The frequency of W-box (TTGAC) elements was identified.

### Analysis of CsWRKY25 Subcellular Localization

The pEAQ-CsWRKY25-GFP and pEAQ-GFP (control) were built and transiently expressed in tobacco leaves to determine the subcellular localization (Deng et al., 2020). The GFP signals were observed with an epifluorescence microscope (Eclipse TS100, Nikon, Japan) after 48 h of infiltration.

### Analysis of CsWRKY25 Transcriptional Activation

We employed a yeast transformation system (Clontech, United States) to test the transcriptional activation activity of CsWRKY25 as described previously (Wang et al., 2021). The vector pGBKT7-CsWRKY25 was built. The fusion construct pGBKT7-CsWRKY25, positive control pGBKT7-53 + pGADT7-T and negative control pGBKT7 were used to transform the yeast cells Gold Y2H. The transformants were cultured on SD/-Trp or SD/-Trp-His-Ade media (Coolaber, Beijing, China).

### Dual-Luciferase Reporter Assays

The transcriptional activity of CsWRKY25 was analyzed with dual-luciferase assay system. The vector pBD-CsWRKY25 and pEAQ-CsWRKY25 was built as the effectors, and promoters of *CsRbohB*, *CsRbohD* and *CsPR10* were cloned into pGreenII 0800-LUC vector as reporters, respectively. The reporter and effector were cotransformed into tobacco leaves to measure the LUC/REN ratio using a dual-luciferase assay kit (Cat No.11402, Yeason, Shanghai, China) at 2–3 days after infiltration (Sainsbury et al., 2009; Fan et al., 2018a; Wang et al., 2021). At least six independent repeats were set.

### Recombinant Protein Preparation and Electrophoretic Mobility Shift Assay

A truncated fragment of the CsWRKY25 (from 211 to 592 aa) C-terminal domain was cloned into the pGEX-6p-1 vector. The GST-CsWRKY25 protein were expressed in Rosetta (DE3) cells and purified using GST resin (Transgen, Beijing, China) (Fan et al., 2018b). Synthesized Oligonucleotide probes containing the W-box element derived from *CsRbohB*, *CsRbohD*, and *CsPR10* promoters were labeled using EMSA Probe Biotin Labeling Kit (Beyotime, Jiangsu, China). Chemiluminescent EMSA Kit (Beyotime, Jiangsu, China) was used to Electrophoretic mobility shift assay (EMSA) according to our previous procedures (Wang et al., 2021).

### Transfection of Citrus Peel by Agroinfiltration

For the transient overexpression assays, pEAQ-CsWRKY25 and PEAQ mentioned above were infiltrated in citrus peel tissue as our previous procedures (Wang et al., 2021). Briefly, two holes (3 mm × 3 mm) were drilled at two sites around the equator of each fruit. *A. tumefaciens* transformants (0.5 mL of 0.5 OD_600_ units) were injected into citrus peel. After 2 h, two new holes that were 1 cm away from the initial holes were drilled, and *P. digitatum* spores (10 μL, 1 × 10^4^ CFU mL^–1^) was inoculated therein. All fruit were stored at room temperature. The incidence of disease and diam of disease spot were measured daily. The incidence of disease (%) = number of diseased fruits/total number of fruits × 100%. The diam of disease spot (mm) = SUM of all diseased fruits [spot length (mm) + spot width (mm)]/total number of fruits/2. Additionally, citrus peel tissue that were infiltrated with only pEAQ-CsWRKY25 or PEAQ were excised for the analyses of enzyme activity, gene expression, H_2_O_2_ content, and lignin content. The peel around the *Agrobacterium* infiltrated holes with a diameter of 2 cm was excised from the fruit and collected, frozen in liquid nitrogen and stored at −80°C. At least three parallel samples for each group were prepared for the measurements.

### Determination of Lignin, H_2_O_2_ Content and Enzymatic Activity

Citrus peel tissue infiltrated with pEAQ-CsWRKY25 plasmids were used for the determination of lignin, H_2_O_2_ content and enzymatic activity. Lignin content was monitored spectrophotometrically at 410 nm, based on the procedure reported by Cai et al. (2006). H_2_O_2_ concentration was obtained by the standard curve according to He’s method He et al. (2013) with minor modifications. Catalase, cinnamyl alcohol dehydrogenase, phenylalanine ammonia-lyase, and peroxidase activities were determined with the methods adapted from previous reports Enzymatic activities are expressed on a fresh weight basis (Goffner et al., 1992; Choudhary et al., 2017; Li et al., 2019).

### Data Analysis

Students *t*-test was used for statistical analysis at *P* < 0.05 (*) or *P* < 0.01 (^**^). Data were presented as means ± their standard errors (SEs) from at least three or six biological repeats calculated by GraphPad Prism 7 software (GraphPad Software, United States). All primers were listed in Supplementary Table 1.

## Results

### Identification and Molecular Characterization of CsWRKY25

**FIGURE 1:**
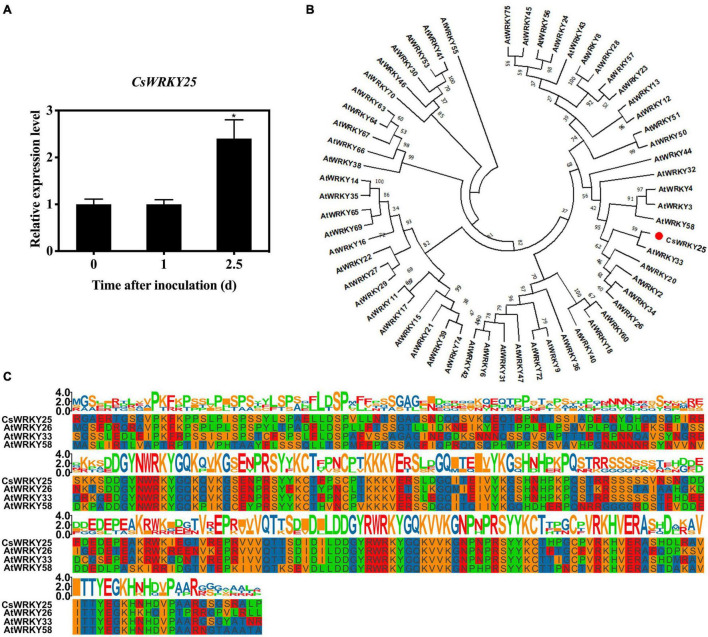
Sequence and phylogenetic analyses of CsWRKY25. **(A)** The relative expression levels of *CsWRKY25* in *P. digitatum* infected citrus peel. Values equal the mean ± SE (*n* = 3). **(B)** The phylogenetic tree of CsWRKY25 and WRKYs from *Arabidopsis thaliana*. **(C)** Multiple alignment of CsWRKY25 with *Arabidopsis thaliana* AtWRKY26, AtWRKY33 and AtWRKY58. *P* values were regarded as statistically significant at *P < 0.05.

In our previous study, *CsWRKY25* (LOC102621617) was found to be up-regulated in transcriptome data of citrus infected by *P. digitatum* (log_2_ fold change = 2.0145), suggesting that CsWRKY25 might be related to the resistance of citrus to *P. digitatum* (Deng et al., 2018). Therefore, the *CsWRKY25* expression of citrus infected by *P. digitatum* was verified by quantitative RT-PCR in this study. As displayed in Figure 1A, the expression of *CsWRKY25* increased remarkably after 2.5 days of infection (*P* < 0.05).

For the analysis of molecular characteristics of CsWRKY25, the open-reading-frame (ORF) of 1,779 bp, was cloned and identified. Its theoretical isoelectric point and predicted molecular weight were 7.59, 64.67 kDa, respectively. The sequence-based homology showed that CsWRKY25 was highly identical to *Arabidopsis thaliana* AtWRKY26, AtWRKY33 and AtWRKY58. The unrooted neighbor joining (NJ) phylogenetic tree constructed by WRKYs from *Arabidopsis thaliana* (Figure 1B; Tamura et al., 2011) suggested that CsWRKY25 was aggregated in Group I WRKYs. Besides, Multiple protein sequence alignments among CsWRKY25 and homeotic WRKYs (AtWRKY26, AtWRKY33 and AtWRKY58) showed that CsWRKY25 possessed two highly conserved WRKY domains (Figure 1C).

### CsWRKY25 Was Localized to the Nucleus and Acted as a Transcriptional Activator

**FIGURE 2:**
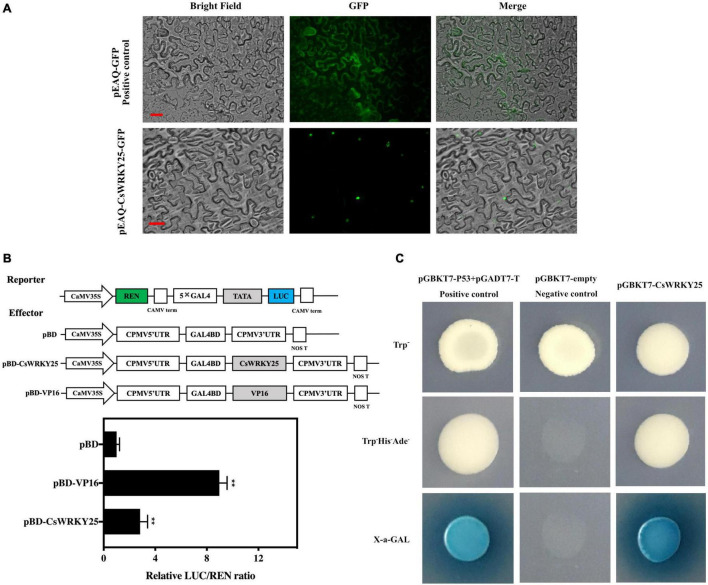
Molecular Characterization of CsWRKY25. **(A)** Subcellular localization of CsWRKY25 in tobacco cells. Bars represent 25 μm. **(B)** *Trans*-activation of CsWRKY25 in tobacco leaves. The ratio of LUC/REN of pBD control was used as calibrator (set as 1) and the pBD-VP16 was used as positive control. Values equal the mean ± SE (*n* = 6). **(C)** Transcriptional activation of CsWRKY25 detected with the GAL4 yeast expression system. *P* values were regarded as statistically significant at **P < 0.01.

The subcellular localization of CsWRKY25 was deduced by transiently expressing a transgene comprising the *CsWRKY25* coding sequence fused to GFP in tobacco cells. The pEAQ-CsWRKY25-GFP signal was predominantly localized in the nucleus, whereas as in the control the pEAQ-GFP signal could be visualized in both nucleus and cytoplasm (Figure 2A), indicating that CsWRKY25 protein was located in the nucleus. To verify the transcriptional activity of CsWRKY25 protein, both the dual-luciferase reporter system and yeast transformation system were employed. The results showed that CsWRKY25 remarkably increased the activity of LUC reporter expression (*P* < 0.01) (Figure 2B) when compared with the empty control pBD. In addition, CsWRKY25 and positive control yeast cells grew better on the SD/-Trp-His-Ade media and expressed X-α-gal activity (Figure 2C). Briefly, CsWRKY25 was a transcriptional activator localized in the nucleus.

### CsWRKY25 Activated *CsRbohB*, *CsRbohD*, and *CsPR10* Expression

**FIGURE 3:**
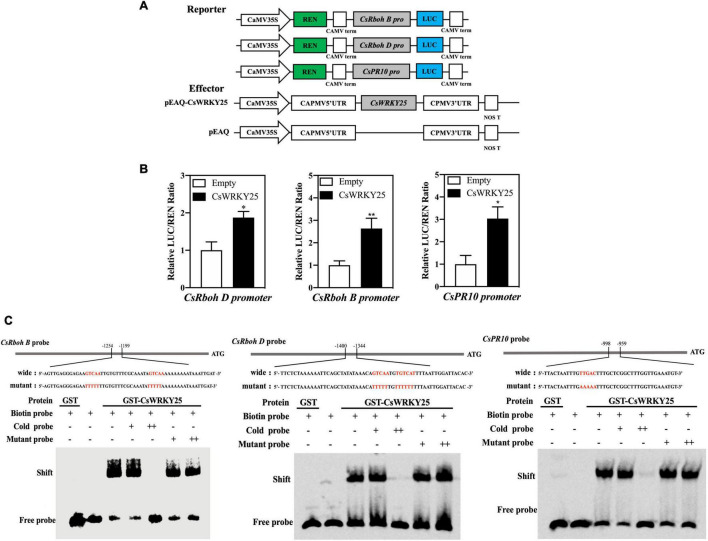
The effects of CsWRKY25 on the transcriptions of *CsRbohB*, *CsRbohD*, and *CsPR10* by binding to W-box. **(A)** Diagram of the vector construction used in the dual-luciferase transient expression assay. **(B)** The transcriptions of *CsRbohB, CsRbohD*, and *CsPR10* were activated by CsWRKY25. Values equal the mean ± SE (*n* = 6). **(C)** GST-CsWRKY25 binded to the *CsRbohB*, *CsRbohD* and *CsPR10* promoters *via* the W-box element. Cold probe (unlabeled probe) is used as the competitor. “−”, “+” and “+ +” represent absence, presence or 100-fold amounts of cold or mutant probe, respectively. The sequences of the wild-type or mutated probes are labeled with biotin, and W-boxes are shown using red letters. *P* values were regarded as statistically significant at *P < 0.05, **P < 0.01.

Immunity related genes were induced to be up-regulated when citrus fruit was infected with *P. digitatum* (Deng et al., 2018). This study indicated that the promoters of *CsRbohB, CsRbohD*, and *CsPR10* contained W-box elements (Supplementary Text 1). To examine the *trans*-activation of CsWRKY25 on *CsRbohB, CsRbohD*, and *CsPR10*, dual-luciferase report assay was also used. Figures 3A,B presented the transcription activation of CsWRKY25 by the ratio of LUC to REN, respectively. With the absence of CsWRKY25 (empty), the ratio of LUC/REN was low, while with the presence of CsWRKY25, the ratio of LUC/REN was significantly increased. These data inferred that CsWRKY25 activated the transcription of *CsRbohB*, *CsRbohD*, and *CsPR10*.

The direct binding of CsWRKY25 to immunity related promoters was determined by EMSA, and the recombinant GST-CsWRKY25-C fusion proteins were purified. Results revealed that the GST-CsWRKY25 protein was successfully bound to the W-box of *CsRbohB*, *CsRbohD*, and *CsPR10* promoters. With the increased amount of cold probe, the binding was clearly decreased, whereas the mutant competitor (AAAA/TTTT) did not cause a decrease in the binding. In addition, as a negative control, the shift bands was abolished when biotin-labeled probes were incubated with GST protein only (Figure 3C). Taken together, it indicated that the binding and activation of CsWRKY25 to the three genes promoters was sequence-specific.

### Transient Overexpression of *CsWRKY25* Enhanced Resistance to *P. digitatum* in the Peel Citrus Fruit

**FIGURE 4:**
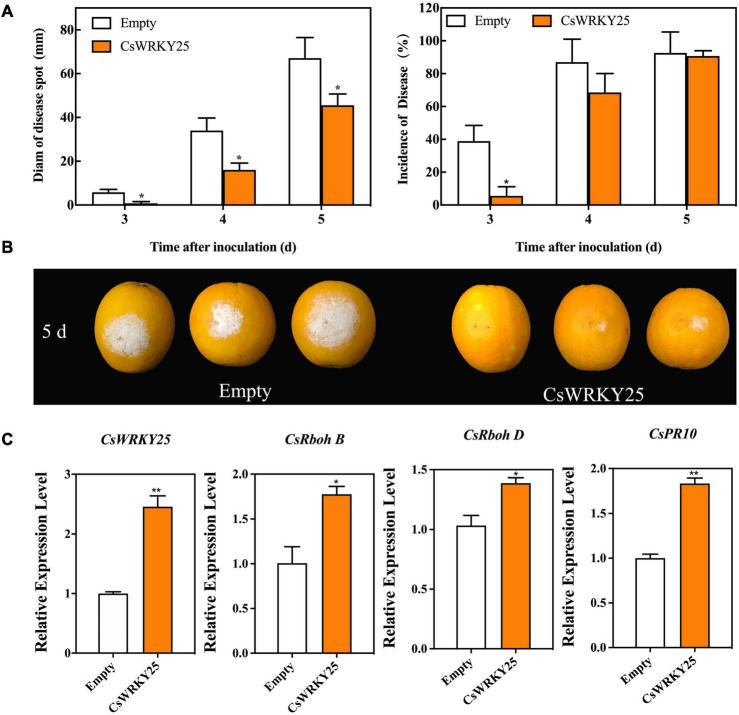
Effects of transient overexpression of *CsWRKY25* on resistance to *P. digitatum* in citrus fruit. **(A)** Diam of disease spot and incidence of disease affected by *CsWRKY25* transient overexpression. **(B)** Phenotype of citrus fruit at 5 days after inoculation. **(C)** The relative expression levels of *CsWRKY25, CsRbohB, CsRbohD*, and *CsPR10*. *P* values were regarded as statistically significant at *P < 0.05, **P < 0.01.

For perennial woody plant, such as citrus, genetic transformation is difficult and time-consuming due to the technical limitations. To obtain more evidence of biological function of CsWRKY25, the transient expression assays of the gene were performed *via Agrobacterium*-mediated transformation of citrus peel *in planta*. As shown in Figures 4A,B, the disease spot diam of CsWRKY25 group was significantly smaller than that in empty group after 3, 4, and 5 days of inoculation (*P* < 0.05), revealing that *CsWRKY25* overexpression enhanced the resistance to *P. digitatum* of citrus fruit. In addition, the expression levels of *CsRbohB, CsRbohD*, and *CsPR10* was upregulated with the overexpression of *CsWRKY25* (Figure 4C).

### CsWRKY25 Induced H_2_O_2_ and Lignin Accumulation in Citrus Peel

**FIGURE 5:**
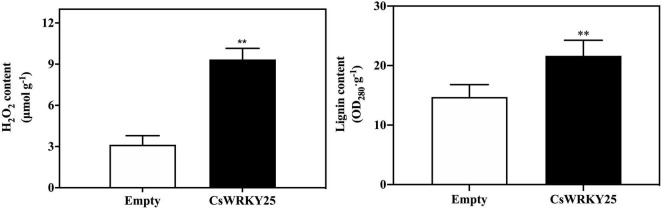
Hydrogen peroxide (H_2_O_2_) and lignin content in citrus fruit peel with the transient overexpression of *CsWRKY25*. The H_2_O_2_ and lignin content are determined by fresh weight. Values equal the mean ± SE. *P* values were regarded as statistically significant at **P < 0.01.

H_2_O_2_ levels were examined for 2 days after infiltration, since H_2_O_2_ is a critical mediator of an early hypersensitive responses (HR) (Pitino et al., 2015). And the lignin levels were also assessed to evaluate the effects of lignification of citrus fruit peel when *CsWRKY25* was transiently overexpressed. Results showed that *CsWRKY25*-transformed citrus fruit peel showed more H_2_O_2_ and lignin accumulation than empty group (Figure 5). Briefly, these data implied the lignification might be a mechanism of CsWRKY25 to increase the resistance to *P. digitatum.*

### *CsWRKY25* Overexpression Activated the Enzymatic Antioxidant System and Established ROS Homeostasis

**FIGURE 6:**
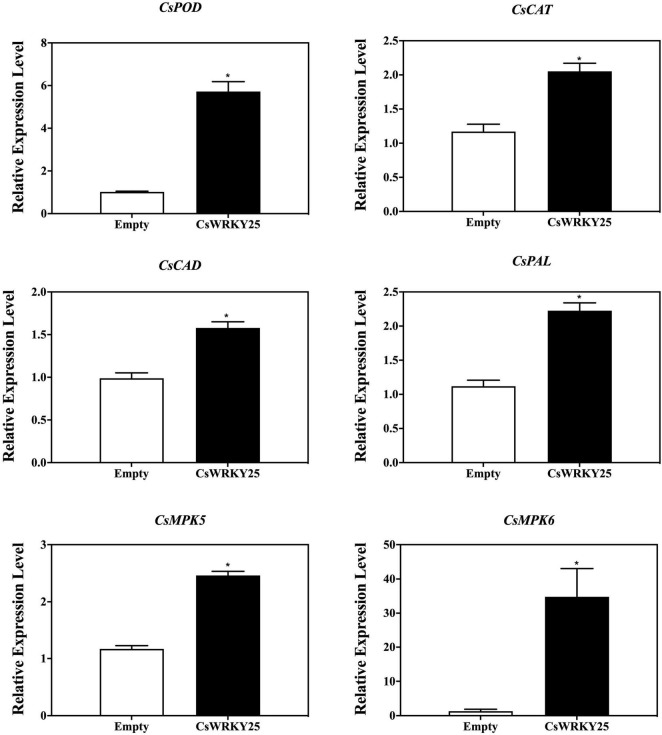
Transcript levels of *CsPOD, CsCAT, CsCAD, CsPAL, CsMPK5*, and *CsMPK6* in pEAQ and *CsWRKY25* overexpressed citrus fruit peel. *P* values were regarded as statistically significant at *P < 0.05.

**FIGURE 7:**
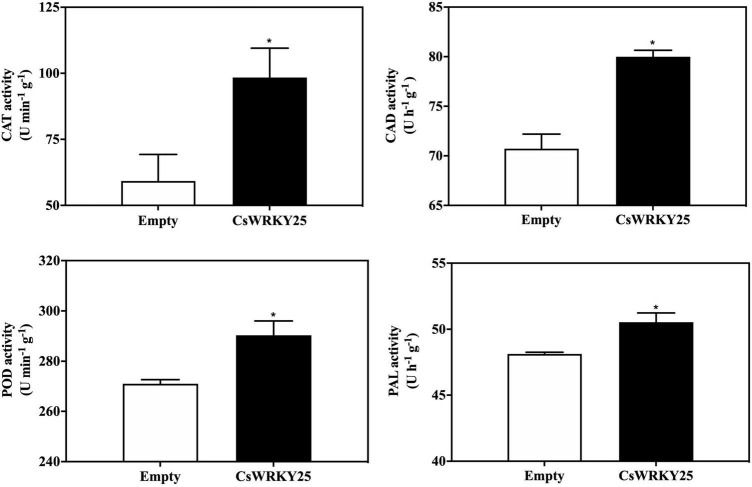
Catalase (CAT), cinnamyl alcohol dehydrogenase (CAD), phenylalanine ammonia-lyase (PAL), and peroxidase (POD) activities in pEAQ and *CsWRKY25* overexpressed citrus fruit peel. Enzymatic activities are expressed on a fresh weight basis. *P* values were regarded as statistically significant at *P < 0.05.

Low levels of ROS is harmless, but high amounts of ROS may cause oxidative damages in plant cells. And the ROS homeostasis in plants is maintained through the ROS enzymes system, especially SOD, POD and CAT (Du et al., 2001). Therefore, the gene expression of these enzymes (*CsPOD, CsCAT, CsCAD*, and *CsPAL*) were detected as well as their enzymatic activity in *CsWRKY25* overexpressed citrus fruit peel. The expression levels of *CsPOD, CsCAT, CsCAD*, and *CsPAL* were increased in *CsWRKY25* overexpressed citrus fruit peel (*P* < 0.05) (Figure 6). Besides, *CsMPK5* and *CsMPK6*, the gene encoding MAP kinase (mitogen-activated protein kinase, MAPK), were upregulated in the *CsWRKY25* overexpressed citrus fruit peel, when compared with empty group without *P. digitatum* infection. Specially, *CsMPK6* were markedly upregulated, with more than 34-fold increases in gene expression. For the enzymatic activity assay, the activities of CAT, CAD, POD, and PAL were upregulated in *CsWRKY25* overexpressed citrus fruit peel when compared with empty group (Figure 7). Since the levels of POD, CAT and PAL activity are indicative of plant disease resistance, the increased disease resistance in citrus caused by overexpression of *CsWRKY25* may be attributed to the accumulation of ROS, which activates the antioxidant defense.

## Discussion

Since citrus is considered as one of the most important economic fruit around the world, it is significance to explore the role of WRKY TFs in citrus disease resistance and the regulatory mechanisms therein. Although the disease resistance mechanism of WRKYs have been studied in various species of plants, the possible regulatory mechanism and the effects on downstream target genes are mostly unclear. And the molecular information of WRKY for initiating defense responses against pathogen in non-model woody perennial species, such as citrus, were limited. Our previous results showed that CsWRKY70 is involved in mediating the endogenous SA signaling pathway, (Deng et al., 2020), CsWRKY65 is involved in mediating the accumulation of ROS and regulating the *PR10*, *CDPK33* gene (Wang et al., 2021), which enhance disease resistance in citrus fruits. This also suggests that many transcription factors are participating together in the regulation of several disease resistance pathways, which is a complex network, and the interactions of these transcription factors need to be further elucidated. The infection of *P. digitatum* can cause the response of *CsWRKY25* in citrus fruit peel. The molecular characteristics of CsWRKY25 showed a high percent identity to AtWRKY26, AtWRKY33 and AtWRKY58, and it was clustered in Group I WRKYs (Figure 1). AtWRKY25, AtWRKY26 and AtWRKY33 were related to thermotolerance of *Arabidopsis* (Li et al., 2011). For their roles on disease resistance, the AtWRKY58 was identified as the direct target of *AtNPR1* (Wang et al., 2006). AtWRKY33 has been widely investigated and confirmed to perform a critical role in immune system of plants in response to necrotrophic pathogens (Lai et al., 2011; Zhou et al., 2015). AtWRKY33 also has been proved as a modulator for plant hormone signaling (Birkenbihl et al., 2012). The high identity of CsWRKY25 and these genes inferred the role of CsWRKY25 in improving citrus resistance.

CsWRKY25 functioned as a transcriptional activator in the nucleus (Figure 2), indicating that CsWRKY25 was able to activate downstream target genes. Furthermore, we found that the *CsRbohB, CsRbohD*, and *CsPR10* promoters contained functional W-box *cis*-element (Supplementary Text 1). By using DLR and EMSA assays, Figure 3 revealed that CsWRKY25 could activate *CsRbohB, CsRbohD*, and *CsPR10* expression. NADPH oxidase enzymes encoded by the *Rboh* gene could cause the accumulation of ROS, which is critical in the defense system of plants, because it can inhibit the pathogens infection process by directly inhibiting pathogen or inducing hypersensitivity at the infection site (Torres and Dangl, 2005; Lehmann et al., 2015; Camejo et al., 2016; Segal and Wilson, 2018). And as a signal molecule, it participates in the strengthening of cell wall and promotes pathogenesis-related genes (*PRs*) (Liu et al., 2021).

Multiple WRKYs silencing can compromise the upregulation of *RbohB*, leading to a restricted ROS bursts in *Arabidopsi* (Adachi et al., 2015). In addition, BnaWGR1, a WRKY TF from rape oilseed, accelerated leaf senescence by promoting the expression of *RbohD* and *RbohF* (Yang et al., 2018). PR proteins are produced after infection in plants, and a number of researches have investigated that WRKYs could regulate the expression of *PR* genes. For example, OsWRKY6, a WRKY TF from rice, could activate the expression of *OsPR10a* (Choi et al., 2015). Therefore, CsWRKY25 may enhance citrus disease resistance through the regulation of *CsRbohB, CsRbohD*, and *CsPR10.* Since it is difficult and time-consuming to conduct the stable transformation in citrus fruit, we performed *Agrobacterium*-mediated transient overexpression *CsWRKY25* in the peel of citrus fruit. Our RT-qPCR validation confirmed the success of the transient overexpression, and illustrated that the transient overexpression of *CsWRKY25* up-regulated the *CsWRKY25*, *CsRbohB, CsRbohD*, and *CsPR10* expression in citrus peel (Figure 4C). In addition, transient overexpression of *CsWRKY25* enhanced disease resistance against *P. digitatum* (Figures 4A,B), and promote the accumulation the hydrogen peroxide and lignin (Figure 5), that also resulted in significant overexpression of *CAT*, *CAD*, *POD*, and *PAL* genes (Figure 6), and resulted in increased activity of the enzymes they encode (Figure 7). ROS is an important signaling molecule in plant immunity, and has the function to strengthen the cell wall. Lignin, a major component of the plant cell wall, could strengthen the cell wall structure (Jung and Deetz, 2015). And cell wall lignification is particularly important during the plant defense against pathogens (Chang et al., 2015). The accumulation of ROS can also activate the plant antioxidant system to maintain the homeostasis of ROS. Wherein, CAT, SOD and POD are the key enzymes to scavenge ROS in plant (Wan et al., 2007). And PAL, CAD, and POD are the crucial enzymes in lignin biosynthesis, and they are indicators of plant disease resistance. Beyond that, we found *MPK5* and *MPK6* genes were up-regulated in *CsWRKY25* overexpressed samples (Figure 6). MAPK cascades play crucial roles in regulating plant stress responses, for example, they are important mediators of antioxidant defense (Zhang et al., 2012; Takáč et al., 2016). Furthermore, Group I WRKYs can be triggered by MAPK-dependent phosphorylation, suggesting that they are involved in plant immune response (Ishihama and Yoshioka, 2012). AtWRKY33 was also determined to be a molecular substrate for MPK3/MPK6 (Mao et al., 2011; Jiang et al., 2017). It could be supposed that the regulatory role of CsWRKY25 is related to the phosphorylation process, and the phosphorylation affects its function and the involved mechanisms require further analysis.

## Conclusion

In summary, we proposed that CsWRKY25 activated *CsRbohB, CsRbohD*, and *CsPR10* in citrus peels, causing the burst of ROS, activating antioxidant systems and disease resistance responses, and thereby reducing the disease incidence after *P. digitatum* infection. However, due to the complicated ROS network, further experiments are necessary to investigate the detailed mechanisms. These findings provide new clues for the understanding of the regulation mechanisms that related to the disease resistance of postharvest citrus fruit.

## Data Availability Statement

The original contributions presented in the study are included in the article/Supplementary Material, further inquiries can be directed to the corresponding author/s.

## Author Contributions

KZ conceived and supervised the project. WW designed the experiments, analyzed the data, and wrote the manuscript. WW, TL, and QC performed most of the experiments. KZ, SY, and LD gave advises and edited the manuscript. All authors read and approved the final manuscript.

## Conflict of Interest

The authors declare that the research was conducted in the absence of any commercial or financial relationships that could be construed as a potential conflict of interest.

## Publisher’s Note

All claims expressed in this article are solely those of the authors and do not necessarily represent those of their affiliated organizations, or those of the publisher, the editors and the reviewers. Any product that may be evaluated in this article, or claim that may be made by its manufacturer, is not guaranteed or endorsed by the publisher.
