# Supplementary material for: CsWRKY25 Improves Resistance of Citrus Fruit to Penicillium digitatum via Modulating Reactive Oxygen Species Production
Source: Front Plant Sci. 2022 Jan 10;12:818198. doi: 10.3389/fpls.2021.818198 (PMC8784754; doi:10.3389/fpls.2021.818198)
Supplement: Supplementary file 1 [file Table_1.DOCX]

Supplementary Material

**Supplementary Table S1.** Summary of primers used in this study

| Assay | Primer sequence |
| --- | --- |
| Full length cloning | *CsWRKY25-For*: ATGGCCTCTTCTTCTGGTAACT |
|  | *CsWRKY25-Rev*: TCAAAATAGCAATGACTCGAAGA |
| Subcellular localization | *CsWRKY25-pEAQ-GFP-For:* caaattcgcgaccggtATGGCCTCTTCTTCTGGTAACT |
|  | *CsWRKY25-pEAQ-GFP-Rev:* tgctagtcataccggtAAATAGCAATGACTCGAAGA |
| Y2H | *CsWRKY25-pGBKT7-For:* aggccgaattcccggggatccATGGCCTCTTCTTCTGGTAACT |
|  | *CsWRKY25-pGBKT7-Rev:* ctagttatgcggccgctgcagAAATAGCAATGACTCGAAGA |
| EMSA | *CsWRKY25-pGEX-6p-1-For: CAGGGGCCCCTGGGATCCATGAACAGTGGGTTCCAATCAGAT* |
|  | *CsWRKY25-pGEX-6p-1-Rev: GAGTCGACCCGGGAATTCAAATAGCAATGACTCGAAGA* |
|  | *CsRbohD*-Probe-For: TTCTCTAAAAAATTCAGCTATATAAACAGTCAATGTGTCATTTTAATTGGATTACAC |
|  | CsRbohD-Mutant-For: TTCTCTAAAAAATTCAGCTATATAAACATTTTTTGTTTTTTTTTAATTGGATTACAC |
|  | CsRbohD-Probe-Rev: GTGTAATCCAATTAAAATGACACATTGACTGTTTATATAGCTGAATTTTTTAGAGAA |
|  | CsRbohD-Mutant-Rev: GTGTAATCCAATTAAAAAAAAACAAAAAATGTTTATATAGCTGAATTTTTTAGAGAA |
|  | *CsRbohB*-Probe-For: AGTTGAGGGAGAAGTCAATTGTGTTTCGCAAATAGTCAAAAAAAAAATAAATTGAT |
|  | *CsRbohB*-Mutant-For: AGTTGAGGGAGAATTTTTTTGTGTTTCGCAAATATTTTTAAAAAAAATAAATTGAT |
|  | *CsRbohB*-Probe-Rev: ATCAATTTATTTTTTTTTTGACTATTTGCGAAACACAATTGACTTCTCCCTCAACT |
|  | *CsRbohB*-Mutant-Rev: ATCAATTTATTTTTTTTTAAAAAATTTGCGAAACACAATAAAAATCTCCCTCAACT |
|  | *CsPR10*-Probe-For: TTACTAATTTGTTGACTTTGCTCGGCTTTGGTTGAAATGT |
|  | *CsPR10*-Mutant-For: TTACTAATTTGAAAAATTTGCTCGGCTTTGGTTGAAATGT |
|  | *CsPR10*-Probe-Rev: ACATTTCAACCAAAGCCGAGCAAAGTCAACAAATTAGTAA |
|  | *CsPR10*-Mutant-Rev: ACATTTCAACCAAAGCCGAGCAAATTTTTCAAATTAGTAA |
| DLR | *CsWRKY25-pEAQ-For: caaattcgcgaccggt*ATGGCCTCTTCTTCTGGTAACT |
|  | *CsWRKY25-pEAQ-Rev: agttaaaggcctcgag*AAATAGCAATGACTCGAAGA |
|  | *CsWRKY25-pEAQ-pBD-For:* tcgccgaccggtaggcctATGGCCTCTTCTTCTGGTAACT |
|  | *CsWRKY25-pEAQ-pBD-For:* aaccagagttaaaggcctAAATAGCAATGACTCGAAGA |
|  | *CsRbohD*-pro-0800-For: cactatagggcgaattggTTCTCTAAAAAATTCAGCTATATAAACA |
|  | *CsRbohD*-pro-0800-Rev: ttggcgtcttccatggGAAATTGAATGAATGAATGAATGAATAT |
|  | *CsRbohB*-pro-0800-For: cactatagggcgaattggTACAATAACCAATCATCAATACGCATGATGTGTG |
|  | *CsRbohB*-pro-0800-Rev: ttggcgtcttccatggTTATTACGTCGATCCTGTAAAGAAAAAACAAAGC |
|  | *CsPR10*-pro-0800-For: cactatagggcgaattggTTACTAATTTGTTGACTTTGCTCGGCTT |
|  | *CsPR10*-pro -0800-Rev: ttggcgtcttccatggGATATCTGAAAAATGGGAATTGCTTT |
| RT-qPCR | *CsWRKY25-For:* CACCCACCATGGATCTTCCC |
|  | *CsWRKY25-Rev*: AATTGCGTCTGCTAGCTGGT |
|  | *CsActin-**For:* TGGATTCTGGTGATGGTGTG |
|  | *CsActin-Rev:* GTTCGGCTGTGGTGGTAAAC |
|  | *CsRbohB-For*^1^*:* CCAGCTTTGGTCAATGCATAGGGATGAACG |
|  | *CsRbohB-Rev*^1^*:* AAGCCTCTCCTACGAGCCAATGCATC |
|  | *CsRbohD-For*^1^*:* GCCATCACGACCTCCGACGGCCTT |
|  | *CsRbohD-Rev*^1^*:* AGCTCTCGTCGGAAATCTGGTCCCAA |
|  | *CsPR10-For:* GTCTCCCCGTCGAGAATGTT |
|  | *CsPR10-Rev:* CGCATCGACCCTATGCTTTG |
|  | *CsPOD-For*^2^: ACAGGCACTGCTGGACAAAT |
|  | *CsPOD-Rev*^2^*:* AAATGCAGGGCCAGTAGACC |
|  | *CsCAT-**For*^2^*:* CTGCCAGTTCTTTCAACGCC |
|  | *CsCAT-Rev*^2^*:* AAACCCTTAGCACTGGCTCC |
|  | *CsCAD-For:* GTGACAGAGGTCGGAAGCAA |
|  | *CsCAD -Rev:* CTCAAGATCAATGGCGCAGC |
|  | *CsPAL-For*^3^*:* CTCGGCCCTCAGATCGAA |
|  | *CsPAL-Rev*^3^*:* CCGAGTTGATCTCCCGTTCA |
|  | *CsMPK5-For:* TAGGGAGGTTTCCGTTTGCC |
|  | *CsMPK5-Rev:* CCCTAAACTCCCTCGATGCC |
|  | *CsMPK6-For:* GGCATTTTGGTTGGCAGCTT |
|  | *CsMPK6-Rev:* TCCGTTTGATTACAGCAGCCT |

Primer sequence obtained from:

1 Mei, P., Song, Z., & Zhou, C. (2019). Functional study of Csrbohs in defence response against Xanthomonas citri ssp. citri. Functional Plant Biology, 46(6), 543-554.

2 Geng, J., & Liu, J. H. (2018). The transcription factor CsbHLH18 of sweet orange functions in modulation of cold tolerance and homeostasis of reactive oxygen species by regulating the antioxidant gene. Journal of experimental botany, 69(10), 2677-2692.

3 Shi, Q., Febres, V. J., Jones, J. B., & Moore, G. A. (2015). Responsiveness of different citrus genotypes to the X anthomonas citri ssp. citri‐derived pathogen‐associated molecular pattern (PAMP) flg22 correlates with resistance to citrus canker. Molecular plant pathology, 16(5), 507-520.

**Supplementary Text S1.** Nucleotide sequences of the promoter of *CsRbohB, CsRbohD,* and *CsPR10.*

W-box (TTGAC) is indicated in box. Translation start site (ATG) is shown in red.

*>CsRbohB* (XM_006472017.3) promoter

TACAATAACCAATCATCAATACGCATGATGTGTGTGGAAAAAAAAAAAATGAATGGGTTATAGAATGTTTTTTTTTTCTCC**TTGAC**GTGTGTGCTTGAGACATCTGAAAGGACTCTTTAAAATGTAATTGATAGCTGTGATGAAGTTGAGGGAGAAGTCAATTGTGTTTCGCAAATAGTCAAAAAAAAAATAAATTGATTAAATACTAAATATTAAAACGTTTTATCTTGAGGATTACAGATTTCCAGTGGGGTGATGCCAGATGTCGTCGAGAGACGCCATCATCAATTAACAAATTCTTCAACTACTTCAGAAGATAAATTTCATTATATACAATTGAATTAAATCTATTGATGGCGCAGGTAGGCAGGGACAGATATTGAAAAATAATAATTAAGAAAAGATAAGGGTTTTAATCGGAATTTAATTAATTTCTTCGTTAATTTTATGTTGTGACCCCTATAAATTTACAGATTTTGTATTATAGCATCATAAAGTTTGTAGTTTCTTCATTTTGCTCTCCATTGAACCAAAATCCTGTTCTAATGGCGTGAAATACTAACTTAATTGTTTGTTTTTGTTCTCTTTTGATTTAGTTCCTAAGACTGTAATATTGATAAAGTATTTTACTCTTACATCAAA**TTGAC**AAATTGTTGTTAGTATTCTATTTCACATAAATTTTCAAAAATTTTGAAAACTACGGTAGGGTTTGTTTTCTTAAATCAAATATAAAATTCTATCCGTAGATAATTATTAATTTATCAAATACAATTTGCATGCTAGATCAGTAACATAAGTAATTTAATTATTTTAAAATTCAATAGTAACTATCCGCTTAAGATTTTCATAATTTTCTCGT**TTGAC**AAGCAACATTGGGTTGTTGAAATTTAATGGTGATTCA**TTGAC**AAGATGGAATTGGAAACATTGAAAAGTATATGCATAAACAGGCCTAACCACTTTCCACGACTAAGCACTTGAAACAGCCGAAACCCCGCCCCTCGGTTTCTTATCCTTT**TTGAC**TTGTCCCTCCTCACCCAATCCCTCCGTATATATAAACTCTTTCAAATCCTCCTTTCAACACCAATTAAAACTTTCTTGCACAATTAAGCCTCCCACATTTTGCTTTCCTCAGGAAAAAAAGAAAAAAGAAAAAAAAAATCTTCCCACTTTTTTGTCACAAGGTTTGTTAATATTCGTCAGCACTTTTTATCACATTTCCAAGTACTTAATTTTAGTTTTTTCAGTACATAAGAGTTCTTTATAATTCTAGTTTGTTAATAGTGCTAGTTAGTTAAACTGGTAAAACCTGTCAGGATTTTATAAGTAATTTGAAGTTTAAATCGTGCTCGTATATATATGTTAAGCTTTGTTTTTTCTTTACAGGATCGACGTAATAAATG

*>CsRbohD* (XM_006487593.2) promoter

TTCTCTAAAAAATTCAGCTATATAAACAGTCAATGTGTCATTTTAATTGGATTACACAATTAATGAGAGCAAACACAGATTATTTTGTAGATCCTCCCAGAAGGACATCCTAGCCCATTCAAAAACAGTTTCATTTTCATTTTTCTTTATTTTCTCATGAACCAATCTTGATTTGAGCATCATAGTGTTTTTGGAAGGCACTAAACCGATGCTTGGAGCTTTTAGAGCTCAAAGACAATGAAGGATAGATCCGGTCATTTCGCAATCAAGATTTCTTTATCTTGAATTGGCCAAAGCTAAATTTCTTAATCAAAACTAGGGGTGGCAATATAGGTCAAGATTCGTTCATTCGATTCGATTTAATATGAATTCAATGGATTTGGGTTTGAAAAATTAATTTGTTTAATAAATTGGTCAAATTTGATTCGATTTGCTAAATAAACGAATTAAATATGGGATTAAGCTCACAAATTGTTTAAAATTCATTTATTGAACAATGCAATAAAAATAAGAAATCATACAGAAAAGTCACTATGGTTCAAAAATATTTATTTTAAGGATTTTAATTCTTATTATTTGTTTTTTTTAATGGATTGGTAATTTGAAGTTTAGAGCTGTGGAGTTAAGATGTTTAAAACTTTAAATATTGTAAGGATGATAAATCTATGATAGTTTATTTTTCTTAATTTTCGTTGTTTACTAATTATTATTTGATATATAATATGATAATTCTAATTGAATGAATTTAAACAAATCCAAATCTAATTCAATTTATTTATTCATTTGATTAAATGAATAAATGAATATTCGTTTAAAAAACGAATCGATATCTGAATAAGCTATGATTAGTTTAATAAACGAATTGAATTTGAATCTAATTATT**TTGAC**CCAATTCATTTAATATTCAACACGAATTCTTATGAATTCGTTTTGGCCTCCAAATCAAAACCCTTTCAAGTTGAATTGTGAACATTCCAAAAGTCAATGATGAAGCCATAGAATCTTGCTATACATCCTATTTACCAGCAAACAATGATTTCGTATTTTTAGTAACCGTATATTTCCTTTTATATACTATAATTTTTTGTGTTAAAAAAAAAAAAAATGAAAATTTTCTGGTCCTAAGGTCAGGT**TTGAC**CAGCATATCACTTTCCTACAAGCTTCTTCTGAATCACGTTTACATTCAACTTTCTTTATATATATATATATATATATATAAACCATATGATCATCAAATCAGAAATGCATAGACATTTTAAAGAGATTAGACAGCCGCTAACTGTTTGATAATAATCTCTTTTTCTCCATGCAAATATATATCACTCAAAATTAGTAACATACGAGTAGTCTTTCAA**TTGAC**TGAAAATTCTTCATATTCATTCATTCATTCATTCAATTTCATG

*>CsPR10* (XM_006488947.3) promoter

TTACTAATTTG**TTGAC**TTTGCTCGGCTTTGGTTGAAATGTCAATTTGTTCGAAGCTTGAATGTACTCGTGTGTGTTGAAAGTTCATTTCCATAGACCAATCAAACAGCCGTATGACGATATATCATTAATTACTTAGTTATATTAAACTATTATTATCACAGGCAAGGGTCCATTTACAGATATATAAATTAATCGATTGATTTGCTACATACCATTAAACCAAACGGCAGAGTTTTCTTTTTGGATATAATCTATTGATGAGTGAAGTGATTGATTGATAATAAAATTGTCGAAGCATTGATGTAGCCAAAATATTGTTGAATATATAATACAAAGATGCCTTAATTCAAATTATGATCCGCATATGCTCTAGGAAAGAACAAAAATGTCCAATTCGCTTCCTCGTAAAATTTGAATAGAAAGCTGCCCATACCACCATATGTAAATCTTTTTACTGTAATTTCCTAAAGTATTACTATCGCTGTCCGCTGGCAATATATATGTTCAAGGACATTTTTAACTACTGGGTAAATCATTTTCTTGGTCATTAATTTCATGTTTCAGTTCAGCCAGTTCTGTTAATTAATGTGTGTTTTAAGCTTTTTGGAAACATTTTGAACTTTGTTTGTATTTGCCTTGTCTCAGCATGATAACAGAGACCAAGGGCCCTTTCGAGATCTAAAAGGTTTTTTTAAAGCCATTGAAATCCTTAGTTTTGTTTCTTTGCATTTGTGTTAATTTAATCTTCAGGAAGGTTCTCTAGTCTTTGGAAAATCAGCCCACATAATGTCTTGATGCAAAGCAGGAATGCAAAGACAAAATTAAATTTTCATCTTATCATAATGTGTACATAATATATACATGCGCGCCAATTAAAAAAAAATTAAGAACAAAAACAACTCCTACACTTAAAGCTCTATAAATACCAAACCCTCTGACCATATTAATTAAGATTCTTCTCATTGCTAGTAAAAGCAATTCCCATTTTTCAGATATCATG
